# A Rare Desmoglein-2 Gene Mutation in Arrhythmogenic Right Ventricular Cardiomyopathy Inciting Incessant Ventricular Fibrillation

**DOI:** 10.7759/cureus.3388

**Published:** 2018-09-29

**Authors:** Glenmore Lasam, Joshua B Oaks

**Affiliations:** 1 Internal Medicine, Overlook Medical Center, Summit, USA

**Keywords:** arrhythmogenic right ventricular cardiomyopathy, ventricular fibrillation, automatic implantable cardioverter defibrillator, radiofrequency ablation, arrhythmia, desmoglein 2 gene mutation

## Abstract

A case of a 51-year-old female with history of hypertension and a significant family history of premature coronary artery disease presented to the hospital after cardiac arrest. She successfully completed a targeted temperature management therapy with full neurologic recovery. Her hospital course was complicated by several bouts of ventricular fibrillation (VF) arrest which was rescued by timely defibrillation, high quality cardiorespiratory resuscitation, and administration of antiarrhythmic medications and inotropic agents. An automatic implantable cardioverter defibrillator (AICD) was inserted for secondary prevention of sudden cardiac death (SCD). A targeted genetic testing for idiopathic ventricular fibrillation revealed a mutation in the desmoglein-2 (DSG2) gene involved in arrhythmogenic right ventricular cardiomyopathy (ARVC). Eventually, a ventricular fibrillation radiofrequency ablation prevented recurrence of fatal arrhythmia and its associated symptoms.

## Introduction

Arrhythmogenic right ventricular cardiomyopathy (ARVC) is an inherited form of heart disease which affects the right ventricle through gradual myocyte replacement by fibrous tissue. It is an infrequent but progressively identified hereditary cardiomyopathy that has been linked to ventricular arrhythmias and sudden cardiac death (SCD) [1]. SCD attributed to ventricular tachycardia (VT) or ventricular fibrillation (VF) is the leading cause of death due to ARVC, however, it can also be the initial expression of the disease [2] with an annual incidence of 0.08% in family cohort [3] to 2.9% in symptomatic patients [4]. Our vignette describes an incessant ventricular fibrillation as an initial presentation of ARVC which is detrimental that could advance to sudden cardiac death. Management is focused at averting this deleterious outcome through an early and timely intervention to abolish the life-threatening arrhythmias that could result in cardiac arrest.

## Case presentation

**Figure 1:**
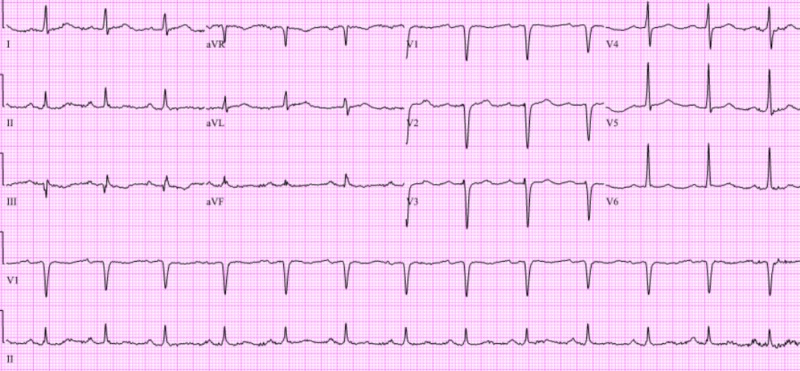
Patient’s initial twelve-lead electrocardiogram in the emergency room after out-of-hospital cardiac arrest. The tracing revealed sinus rhythm with age undetermined septal infarct.

A 51-year-old female with a history of stage 1 hypertension was admitted to the hospital post cardiac arrest. She had a significant family history of premature coronary artery disease. Her father had a coronary artery disease diagnosed in his 40’s, her mother had a pacemaker inserted, and she had two first degree relatives diagnosed with an early cardiac disease. She is a nonsmoker, nonalcoholic drinker and denied recreational drug use. She was in her usual state of health and functionally active until her husband noticed her moaning and eventually unresponsive in the middle of the night. Prior to this, the patient did not complain of any chest pain, dyspnea, dizziness, lightheadedness or any other constitutional symptoms. Her husband started cardiopulmonary resuscitation and was taken over by the emergency medical staff (EMS). She was intubated and defibrillated five times by EMS for ventricular fibrillation then was given a bolus of amiodarone. She eventually had a return of spontaneous circulation and was transported to the hospital in which she was noted to have decorticate posturing with no purposeful movements. Electrocardiogram (ECG) during this time showed sinus rhythm (Figure 1). She was admitted to the coronary care unit and was placed on targeted temperature management and was maintained on amiodarone drip. Transthoracic echocardiogram showed no valvular abnormalities, normal left atrium and left ventricular cavity size but with borderline concentric left ventricular hypertrophy with an ejection fraction of 56% and note of subtle regional wall motion abnormalities. About 12 hours after initiating the targeted temperature management, the patient was in severe bradycardia in the mid 30’s and a decision was made to hold the amiodarone at that point. The patient completed the targeted temperature management protocol and eventually had a full neurologic recovery thereafter.

**Figure 2:**
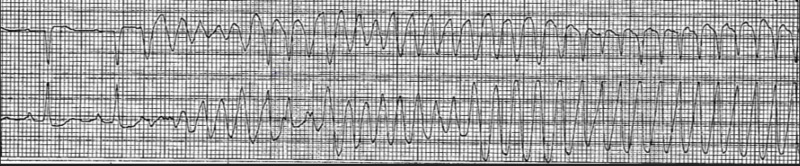
Patient’s telemetry strip on her witnessed cardiac arrest after she neurologically recovered from targeted temperature management. The tracing revealed ventricular fibrillation and ventricular tachycardia.

**Figure 3:**
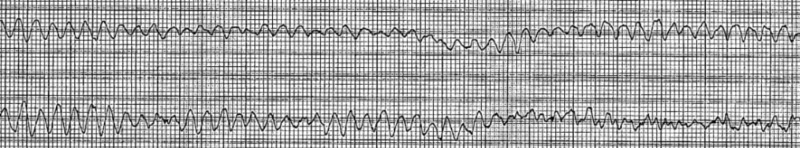
Patient’s telemetry strip during her subsequent cardiac arrest. The tracing revealed ventricular fibrillation.

She was extubated and subsequently transferred to the floor. She was scheduled for cardiac catheterization; however, she had witnessed ventricular fibrillation arrest on the floor (Figure 2). Advanced cardiopulmonary resuscitation was initiated and she was revived immediately after defibrillation. A total of three minutes of resuscitation was performed and she was following commands afterwards. She was also given an amiodarone bolus and transferred back to coronary care unit. ECG did not show any ST elevations at this time. Subsequently, she had 23 more episodes of ventricular fibrillation arrest (Figure 3) over a six-hour period which were intervened by timely defibrillation and administration of antiarrhythmic medications and inotropic agents. The antiarrhythmic medications administered were amiodarone and lidocaine boluses and drips. Intravenous magnesium was also given during the resuscitation course. Emergent cardiac catheterization showed minimal coronary artery disease. A transvenous pacemaker wire was placed due to bradycardia during a code in the catheterization laboratory, although the bradycardia did not initiate the arrest. An intra-aortic balloon pump was placed. Subsequently the balloon pump and temporary pacemaker wire were removed after she eventually became more hemodynamically stable. She once again had a full neurologic recovery and underwent an uneventful implantation of a dual-chamber implantable cardiac defibrillator (ICD). She was discharged from the hospital on amiodarone therapy and was closely followed up in the cardiology outpatient clinic. Cardiac magnetic resonance imaging was done which showed mild biventricular enlargement but with normal regional systolic function, no late gadolinium enhancement consistent with the absence of myocardial inflammation, infiltration or infarction and with no imaging features of ARVC. She eventually had a targeted genetic testing for idiopathic ventricular fibrillation and was found to have a mutation in the desmoglein-2 (DSG2) gene (c.338T > C, pVal1113Ala), which is a gene typically involved in ARVC.

She had been readmitted several times to the hospital due to palpitations, dizziness, lightheadedness, and defibrillator shocks. Her defibrillator had been interrogated as well as her antiarrhythmic medication had been adjusted which improved her symptoms and reduced defibrillator discharge. Because of intermittent episodes of defibrillator firing due to ventricular fibrillation despite antiarrhythmic medications and intracardiac device, a ventricular fibrillation radiofrequency catheter ablation was performed successfully. Thereafter, she had no repeated episodes of palpitations, dizziness, lightheadedness, and defibrillator shocks.

## Discussion

ARVC is transmitted genetically through an autosomal dominant trait that has a variable penetrance of 20–25% [5,6] with the chromosomal locus mapped at 14q23-q24 [7] while a recessive variant called Naxos Disease presents with cardiac dysfunction features of ARVC, palmoplantar keratosis, and woolly hair with the chromosomal locus identified at 17q21 [8]. There are five confirmed disease-inducing genes that encode desmosomal proteins which include plakoglobin (JUP), desmoplakin (DSP), plakophilin-2 (PKP2), desmoglein (DSG), and desmocollin (DSC2) which have been involved in autosomal dominant disease while JUP and DSP have been linked to autosomal recessive disease [9].

The pathogenesis of ARVC had been attributed to a dysfunctional desmosome inducing myocardial detachment and cellular demise as well as myocardial inflammation commencing as a reparative mechanism which consequently culminate in fibrofatty replacement of injured myocytes [10]. DSG is a desmosomal component and desmoglein-2 (DSG2) is a sole isoform expressed in cardiomyocytes which had been associated with ARVC. The DSG2 gene had been analyzed in ARVC patients of Italian descent with negative mutations in the transforming growth factor beta-3 (TGFB3), DSP, and PKP2 genes, and out of the 54 probands studied, DSG2 mutation had been identified in 16.66% of cohort with ventricular arrhythmias documented in all probands that includes sustained VT with left bundle-branch block morphology (5.5%), nonsustained VT (5.5%), and isolated monomorphic premature ventricular contractions (3.7%) [11]. Also, a study of 33 cases of ARVC patients with no mutation in the PKP2 or DSP genes identified 12.1% DSG2 mutation disrupting a highly conserved amino acid within a functional domain of DSG2 inferred to contribute in the development of ARVC [12]. A DSG2 gene sequence analysis of 86 Caucasian ARVC patients with no mutations in the DSP, PKP2, and JUP genes identified eight mutations in nine probands and detected sustained ventricular arrhythmia and family history of sudden death or aborted sudden death in 8% and 66% patients, respectively [13]. In a study of ARVC clinical course and predictors of arrhythmic risk, 24% experienced one or more life-threatening arrhythmic event (LAE) defined as SCD, aborted cardiac arrest, syncopal VT or electrical storm, or cardiovascular mortality and the essential factors linked with an immense risk severe arrhythmias at follow-up were male gender, participation in strenuous exercise following the diagnosis, history of atrial fibrillation, syncope, and hemodynamically tolerated sustained monomorphic ventricular tachycardia [14].

The use of antiarrhythmic drugs in ARVC may diminish symptoms, however, it did not lessen incidence of fatal arrhythmia or cardiovascular mortality [15]. The dearth of survival benefit of antiarrhythmic therapy advocated the utilization of ICD in ARVC patients. Forty-seven percent of patients implanted with ICD for primary prevention of LAE obtained an appropriate shock that abolished the arrhythmia which reinforce the ICD’s life-saving advantage when used appropriately to prevent SCD in high-risk ARVC patients [14]. A meta-analysis of AVRC patients with ICD for either primary or secondary prevention ascertained an annual rate of 0.9% cardiac death, 0.8% noncardiac death, and 0.9% heart transplantation [16]. The 2017 American Heart Association/American College of Cardiology/Heart Rhythm Society guideline for management of patients with ventricular arrhythmias and the prevention of SCD recommended ICD in patients with ARVC and additional marker of increased risk of SCD that includes sudden cardiac arrest, sustained VT, significant ventricular dysfunction with right ventricular ejection fraction or left ventricular ejection fraction ≤35% [17]. Likewise, the 2015 European Society of Cardiology guideline for management of patients with ventricular arrhythmias and the prevention of SCD recommended ICD implantation in patients with a history of aborted SCD and hemodynamically poorly tolerated VT [18].

## Conclusions

Our vignette unraveled the mystery of an incessant episodes of ventricular tachycardia and ventricular fibrillation that lead to cardiac arrest. A DSG2 gene mutation involved in ARVC had been identified though a targeted genetic testing for idiopathic ventricular fibrillation. ARVC should be explored as an etiology of malignant ventricular arrhythmias and treatment should be geared towards AICD placement as the most effective modality to prevent SCD. Also, a future research on the association of DSG2 gene mutation in ARVC should be instituted to ultimately recommend a definitive treatment.
